# Emotional Contagion in Animals: Connections and Applications

**DOI:** 10.3390/ani14202970

**Published:** 2024-10-15

**Authors:** Andrew C. Gallup

**Affiliations:** David S. Olton Behavioral Biology Program, Department of Psychological and Brain Sciences, Johns Hopkins University, Baltimore, MD 21218, USA; a.c.gallup@gmail.com

The automatic spreading of emotions, i.e., emotional contagion, is frequently observed in social species and is a foundational element of human interaction. From an evolutionary perspective, the sharing of internal states may hold adaptive value in promoting synchronization, affiliation, and prosocial behavior within animal groups [1,2]. Manifesting from a perception–action mechanism or mirroring system [3], emotional contagion is considered to be a substructure for more advanced forms of empathy, such as empathic concern and perspective-taking [4]. Therefore, researchers in the fields of neuroscience, psychology, and behavioral biology have shown considerable interest in understanding the mechanisms of this response.

While the comparative study of emotional contagion has advanced significantly in recent years [5], numerous challenges and open questions remain [6]. For example, motor matching and behavioral contagion are often considered evidence of an emotional coupling or empathic response, yet the empirical studies examining this connection are often mixed and inconsistent [7]. In addition, comparative research shows that emotional contagion is linked to certain ecological circumstances and socio-behavioral traits [8], which can result in varying neurophysiological changes and behavioral reactions that align the affective states of individuals across species. Thus, further research is needed to achieve accurate measurement and labeling of the behaviors and phenomenon associated with emotional contagion in different species.

The goal of this Special Issue was to collate the latest research on (1) the psychological and behavioral correlates of emotional contagion, (2) the proximate mechanisms and physiological markers of emotional contagion, (3) the adaptive/functional significance of emotional contagion, (4) new techniques and methodologies for assessing emotional contagion, and (5) challenges and future directions in the study of emotional contagion in animals. Ultimately, the articles published within this Special Issue address some, but not all, of these topics. However, the contributions do showcase a range of approaches to studying emotional contagion in humans and non-human animals, including semi-structured interviews, self-report and survey responses, behavioral observations, and various neurobiological measurements. In addition, the authors of the articles published in this Special Issue come from a diverse array of countries, including the United Kingdom, Malaysia, the United States, China, and Italy.

A total of six articles were published in this Special Issue. The empirical papers include original research across a broad range of topics and disciplines, including how pet owners perceive and construct experiences of empathy from domesticated dogs and cats (Hiestand et al.), the neurological and physiological responses of goats exposed to a slaughter environment (Kumar et al.), the role of empathic concern and gender on interspecific contagious yawning in humans (Gallup & Wozny), the effects of sensory avoidance on the contagion of positive and negative emotions in piglets (Zhang et al.), and the empathy-like behaviors of domesticated dogs in response to unknown humans in distress (Rivera & Meyers-Manor). A stimulating review article concludes the Special Issue by examining the varying emotional and neurohormonal systems involved in social play in humans and non-human animals (Cordoni & Norscia).

While the research published in this Special Issue represents only a modest contribution to the overall literature, collectively, these articles advance our basic understanding of emotional contagion and other empathic responses while offering important applications for improving both animal welfare standards and human mental health and well-being. Moreover, each article offers valuable suggestions for how future research on emotional contagion could be integrated across different disciplines. I am grateful to the Editors at *Animals* for their generous support, and to all the authors and external reviewers for their contributions to this Special Issue.
